# Mastering the D-Band Center of Iron-Series Metal-Based Electrocatalysts for Enhanced Electrocatalytic Water Splitting

**DOI:** 10.3390/ijms232315405

**Published:** 2022-12-06

**Authors:** Jing Hu, Adel Al-Salihy, Bin Zhang, Siwei Li, Ping Xu

**Affiliations:** 1 School of Energy and Environment, Anhui University of Technology, Ma’anshan 243002, China; 2 MIIT Key Laboratory of Critical Materials Technology for New Energy Conversion and Storage, School of Chemistry and Chemical Engineering, Harbin Institute of Technology, Harbin 150001, China; 3 Institute of Industrial Catalysis, School of Chemical Engineering and Technology, Xi’an Jiaotong University, Xi’an 710049, China

**Keywords:** electrocatalysis, iron-series metal-based materials, d-band center, hydrogen evolution reaction, oxygen evolution reaction

## Abstract

The development of non-noble metal-based electrocatalysts with high performance for hydrogen evolution reaction and oxygen evolution reaction is highly desirable in advancing electrocatalytic water-splitting technology but proves to be challenging. One promising way to improve the catalytic activity is to tailor the d-band center. This approach can facilitate the adsorption of intermediates and promote the formation of active species on surfaces. This review summarizes the role and development of the d-band center of materials based on iron-series metals used in electrocatalytic water splitting. It mainly focuses on the influence of the change in the d-band centers of different composites of iron-based materials on the performance of electrocatalysis. First, the iron-series compounds that are commonly used in electrocatalytic water splitting are summarized. Then, the main factors affecting the electrocatalytic performances of these materials are described. Furthermore, the relationships among the above factors and the d-band centers of materials based on iron-series metals and the d-band center theory are introduced. Finally, conclusions and perspectives on remaining challenges and future directions are given. Such information can be helpful for adjusting the active centers of catalysts and improving electrochemical efficiencies in future works.

## 1. Introduction

Hydrogen production through water electrolysis has become a key link that cannot be omitted from the whole production process and has thus become one of the pillars of the future large-scale new energy industry. Electrochemical water splitting for oxygen and hydrogen production and applications is the main pollution-free way to obtain clean hydrogen energy, drive fuel cells, and realize carbon-free emission [1,2,3]. In recent years, the energy conversion efficiency of electric energy has been greatly improved with the rapid development of electrolytic water technology. Moreover, the cost of electrolytic water splitting has also been drastically reduced due to the exploration of catalysts for water electrolysis that are cheap, efficient, stable, easy to prepare, and result in low environmental pollution [4,5,6,7].

Electrochemical water splitting, a powerful technique, involves applying voltage to a system to promote the decomposition of the water molecules adsorbed on the electrode surface to produce hydrogen and oxygen [8,9,10,11,12]. Given that the use of catalysts with high electrocatalytic activity could reduce the applied voltage, selecting the appropriate catalysts can minimize energy consumption to the greatest extent. In other words, the properties of the catalysts directly affect the efficiency of water splitting [13,14].

Until now, the catalysts with outstanding activities for the oxygen evolution reaction (OER) are still mainly based on Ir- and Ru-based materials, and those for the hydrogen evolution reaction (HER) are still mainly based on Pt-based materials [15,16,17,18,19]. The commercial applications of precious metals are severely limited by their expensive cost and scare supply. Therefore, the preparation of non-precious metal-based catalysts that can replace those based on precious metals is one of the most important topics in the field of electrocatalytic water splitting [20,21]. Cheap substitutes for electrolytic water electrode materials have been developed. They mainly include oxides [22,23], hydroxides [24,25], hydroxyl oxides [26,27], phosphates [28,29], and sulfides [30,31]. Their catalytic active centers are generally transition metal atoms and a few alkali metal atoms because the *d* orbitals of transition-state elements with underfilling electrons can accept electrons or electron pairs [32]. Subsequently, the receptor and donor can form intermediates through coordination, so as to reduce the activation energy of the reaction and promote the reaction at lower energy, that is, they act as catalysts for water splitting [33,34,35].

Among various catalysts, iron-series elements, namely iron-, cobalt-, and nickel-based materials, have attracted considerable attention [36,37,38,39,40,41]. On the one hand, these elements are abundant on earth and therefore the corresponding materials are cost-effective and easy-to-manufacture. On the other hand, many kinds of Fe-, Co-, and Ni-based materials exhibit outstanding performance for OER and/or HER. Interestingly, by studying various reports, we find that these kinds of materials have some characteristics and advantages, including unfilled *d*-orbitals that can provide coordination spaces. In other words, tuning the d-band center of iron transition series metal-based materials is a rough strategy for developing electrocatalysts for water splitting. However, there has not been a review about this important topic in electrocatalysis. Therefore, summarizing the results of the research on catalysts based on iron-series metals is necessary and provides clear ideas for research in the future. In this review, we attempt to offer the readers a comprehensive review of the recent progress in the development of descriptors that correlate electrocatalytic activity of the iron-series electrocatalysts with the d-band center.

## 2. Iron-Series Electrocatalysts for Water Splitting

On the basis of the catalytic reaction that mainly occurs on the active surfaces of catalysts, various compounds of iron transition series elements and their corresponding catalytic reactions can be classified as follows:

Nickel oxide: Nickel oxide (NiO) can effectively open the O–H bond of the water that had adsorbed on surfaces to produce adsorbed hydrogen atoms [42]. Therefore, it is conducive to the HER. For example, Qiao’s group prepared NiO nanorods with surfaces that were rich in O-vacancies and showed a low overpotential of ~110 mV to produce the current density of 10 mA cm^−2^ for the HER in alkaline solutions [43]. In addition, many studies have used NiO as catalyst for the OER and also achieved good results [44]. With the deepening of research, NiOOH instead of NiO has been found to be the real catalyst for the OER [45]. In fact, this phenomenon is considered as a method for energy storage in supercapacitors [46]. Therefore, NiO can be used as both the anode and the cathode for overall water splitting.

Cobalt oxide: There are many kinds of cobalt oxides with different atomic ratios such as Co_3_O_4_, CoO, and Co_2_O_3_ [47,48,49]. Although different cobalt oxides have different atomic ratios, similar to those of NiO, the catalytic active sites of cobalt oxides are also mainly cobalt atoms and oxygen vacancies. For example, when CoO is used as the catalyst for HER, O–H is adsorbed to the Co(111) surface, which is rich in oxygen vacancies, and the remaining hydrogen atom is connected to the adjacent oxygen atom, thus forming an intermediate with increased stability. Cai et al. studied the OER properties of Co_3_O_4_ with rich oxygen vacancies and found that when oxygen defects were introduced into single crystalline ultrathin Co_3_O_4_ nanosheets with O-terminated (111) facets under alkaline conditions, the as-prepared defect-rich Co_3_O_4_ nanosheets showed improved OER activity [50]. When oxygen vacancies were introduced, the increase in the electron concentration of the cobalt atoms and the decrease in valence state resulted in interaction with the surrounding oxygen atoms, thus reducing adsorption energy and changing the OER mode of Co_3_O_4_. Meanwhile, oxygen vacancies can also reduce the band gap of Co_3_O_4_, thus increasing the conductivity of Co_3_O and accelerating the kinetics of OER. The application of Co_2_O_3_ in electrolytic water splitting has been less studied than that of the first two compounds, which is likely related to its difficulty in preparation.

Ferric oxide: Iron oxides also have several compounds with different Fe/O ratios, such as Fe_2_O_3_ and Fe_3_O_4_. As for Fe_3_O_4_, it can be regarded as a mixture of Fe_2_O_3_ and FeO, among which, Fe atoms mainly exist in the valence states of Fe^2+^ and Fe^3+^. When Fe_2_O_3_ is used as the electrode material for the OER, iron atoms on the surface of the material would first adsorb water molecules and then form Fe–O–H. This phenomenon shows that the iron oxide still needs to undergo a phase transition to form FeOOH during the OER [51]. Among iron oxides, Fe_3_O_4_ is the most commonly used electrode material for OER. However, due to its low conductivity, it is often combined with other substances or loaded on collectors with high conductivity. For example, Ni-doped Fe_3_O_4_ particles supported on iron foil show good OER properties because the coexistence of Fe^2+^ and Fe^3+^ creates a large number of active sites that are similar to oxygen vacancies [52].

Hydroxide (M hydroxide; M = Fe, Ni, Co): Given that reducibility follows the order of Fe(OH)_3_ > Co(OH)_2_ > Ni(OH)_2_, Ni(OH)_2_ is more suitable and stable for HER than Fe(OH)_3_, which is easily reduced into Fe_2_O_3_, while Fe(OH)_3_ is more stable and suitable for the OER. On the other hand, Co(OH)_2_ can be used as an electrode material for OER, HER, and overall water splitting [53]. During the OER, Co(OH)_2_ is transformed into high-valence cobalt-based compounds, such as Co_3_O_4_ and CoOOH, which acted as the real active materials for the OER [54].

Hydroxyl oxide (M oxyhydroxide; M = Fe, Ni, Co): For many oxides or sulfides in alkaline solution, hydroxyl oxide acts as the real active materials for the OER. For example, during the OER in alkaline solution, Co_9_S_8_ transforms into CoOOH and Ni(OH)_2_ transforms into NiOOH [55,56]. The OER catalytic activities of these three metal hydroxyl oxides follow the order of FeOOH > CoOOH > NiOOH [57]. Importantly, recent studies have found that bimetallic hydroxyl oxides are also important electrocatalytic materials. For example, binary Fe–Co oxyhydroxide, binary Fe–Ni oxyhydroxide, and binary Ni–Co oxyhydroxide have been proven to have excellent electrocatalytic properties [58,59,60]. Bimetallic hydroxyl oxides have good OER performance because the combination of these two substances promotes the gathering of active atoms on the surfaces of the catalyst, thus resulting in a sharp increase in the number of efficient catalytic active sites.

The above analysis indicates that hydroxyl oxides based on iron-series metals show outstanding OER performance and structural stability. At the same time, hydroxides and metal oxides based on iron-series metals often have superior HER performance and structural stability. Moreover, the combination of different hydroxyl oxides based on iron-series metals is helpful for further improving the OER performance of catalysts. The combination of oxides, hydroxides, or hydroxyl oxides based on iron-series metals is helpful for improving the catalytic performance mainly because of their surface oxygen vacancy concentration, exposed catalytic active area, and the conductivity. These factors are also related to the surface structures of the catalysts. The reported catalyst-related factors mainly include enriched oxygen vacancy surfaces [61,62,63,64,65], ion adsorption [66,67], edge effects [68,69,70,71], single-atom catalysts [72,73,74,75,76,77], and high specific surface areas [67,78,79,80,81,82]. These listed factors are common characteristics of high-performance electrocatalysts. However, the relationships between the electronic structure and performance of the catalysts have not yet been explored. The interface properties of materials are mainly determined by their own electronic structures, such as the outer orbital arrangement and the density of states of the atoms. 

The d-band center theory for iron transition series metal-based catalysts with the unfilled 3*d* orbitals of iron-series metal atoms has attracted wide attention in recent years because the energy difference between the d-band center and Fermi level (i.e., *E*_f_ − *E*_c_) can feasibly predict and explain the adsorption of small molecules, including OH* and H*, on the metal surface, and can thus explain the relationships between electronic structures and catalytic performances [83,84].

The d-band center theory is a theoretical model proposed by Nørskov and Hammer in 1995 to explain the adsorption of substances on catalysts [85]. When the adsorbed material forms a bond with the catalyst, the adsorption capacity is mainly affected by the position of the d orbital center of the metal atom of the catalyst. Therefore, the d-band center theory can be reasonably used to explain the relationships between the electronic structure and the adsorption capacity of the catalyst, as well as to reveal the good coordination ability and electrocatalytic performance of the catalyst from the perspective of electronic structures and energy level changes [86]. The d-band centers of the catalyst atoms can be regulated through the incorporation of dopants, vacancies, strains, and heterostructures. Considering the relatively low electrocatalytic water-splitting activity of single iron-series compounds, various efforts have been made to improve electrocatalytic performances through two effective ways: one is to increase the unit activity on each active site (intrinsic), and the other is to increase the number of active sites (extrinsic) [87,88].

## 3. Strategies for Tuning the D-Band Center of Materials Based on Iron-Series Electrocatalysts

### 3.1. Introduction of Defects/Vacancies

Shifting the d-band center by creating defects or improving vacancies is an effective way to regulate electronic and geometric structures because downshifting can aid the active intermediate desorption of active intermediates and upshifting promotes the adsorption of the active intermediates [89,90,91,92], leading to an enhancement in the reactivity of active sites, which significantly facilitates the activity of electrocatalytic water splitting.

**Figure 1:**
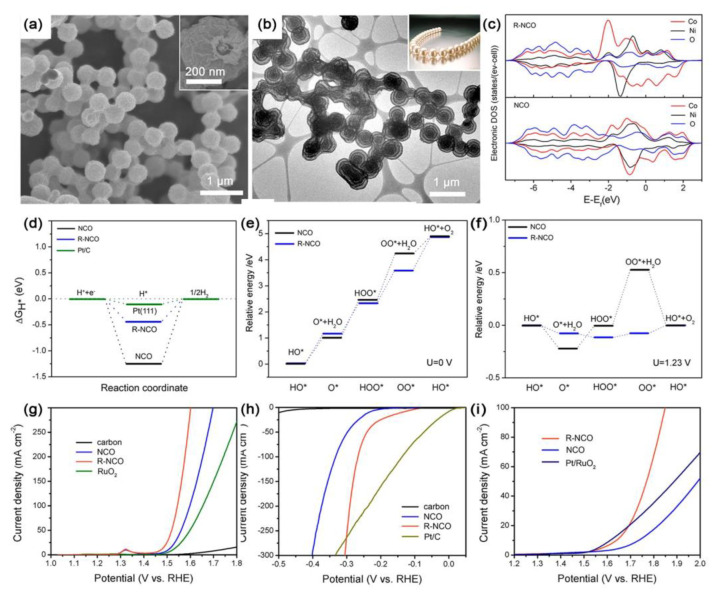
(**a**) SEM image and (**b**) TEM images of R-NCO. (**c**) Calculated DOS curves for pristine NCO and R-NCO. (**d**) Calculated free−energy diagram of the HER on pristine NCO and R-NCO. (**e**,**f**) Schematic illustration of reaction paths for OER on pristine NCO and R-NCO at (**e**) zero potential and (**f**) equilibrium potential at 1.23 V. (**g**) OER, (**h**) HER, and (**i**) overall water-splitting electrocatalytic properties of R−NCO and NCO at a scan rate of 5 mV s^−1^ in 1.0 M KOH. Reproduced with permission from ref. [22]. Copyright 2018, American Chemical Society.

Peng et al. synthesized spinel transitional NiCo_2_O_4_ with a unique necklace-like multi-shelled hollow structure that can offer rich oxygen vacancies (Figure 1a,b), and they found that the introduction of oxygen vacancies through reduction process caused the PDOS of the Co *d* orbital in the reduced NiCo_2_O_4_ to shift towards the low-energy direction and present broadened peaks. The active substances used to compare the d-band center with each other are all compounded with the necklace-like carbon, thus excluding the influence of carbon on the d-band center, which is only affected by the introduction of oxygen vacancies. These changes were indicative of the shifting away of the distribution of electrons in the d-band from the Fermi level (Figure 1c) that thereby increased the spin polarization of Co, lowered adsorption energy (Figure 1d–f), and enhanced the electrocatalytic water-splitting activity of the cobalt site (Figure 1g–i) [22].

**Figure 2:**
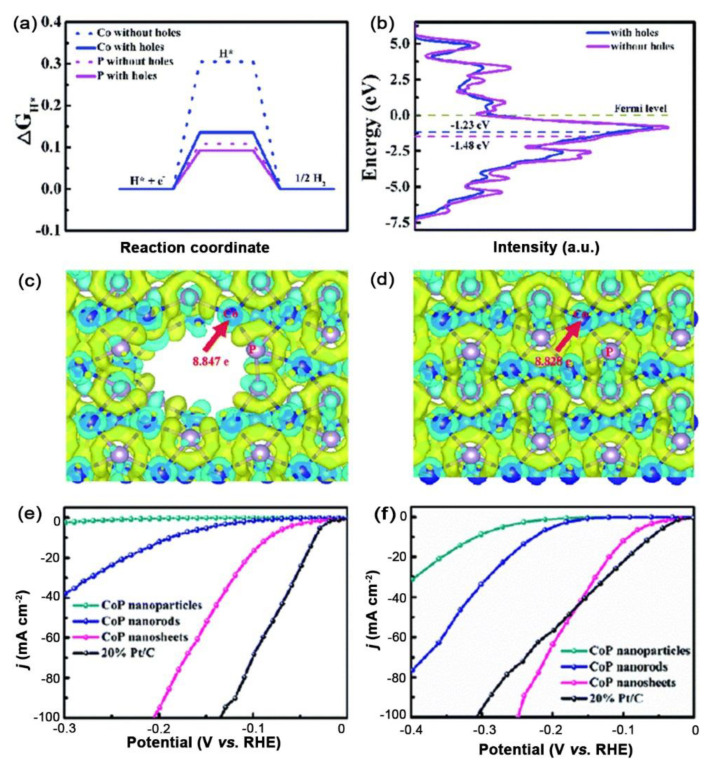
(**a**) The free-energy diagram of Co and P atoms at the top sites of the CoP (011) surface with and without holes, respectively. (**b**) PDOS of CoP with and without holes, respectively. The electron density difference maps of CoP (**c**) with and (**d**) without holes. Yellow and cyan represent electronic accumulation and depletion, respectively. HER polarization curves with a scan rate of 5 mV s^−1^ in (**e**) 0.5 M H_2_SO_4_ and (**f**) 1.0 M KOH. Reproduced with permission from ref. [93]. Copyright 2021, The Royal Society of Chemistry.

In addition, Geng et al. used CoP as a model catalyst to study the actual relationship between holey structures and varied d-band centers [93]. They found that the hole-creating method can successfully tune the d-band center, leading to the upward shift of the d-band center and resulting in an enhanced interaction between hydrogen and cobalt atoms that was beneficial for obtaining the optimal ΔG_H*_ value (close to 0 eV) of CoP (Figure 2a–d). The optimized hydrogen adsorption/desorption behavior accounted for the high performance of the hole-rich CoP with the enhanced pH-universal HER activity of only 84 and 94 mV at the current density of 10 mA cm^−2^ in acidic and alkaline solutions (Figure 2e,f).

**Figure 3:**
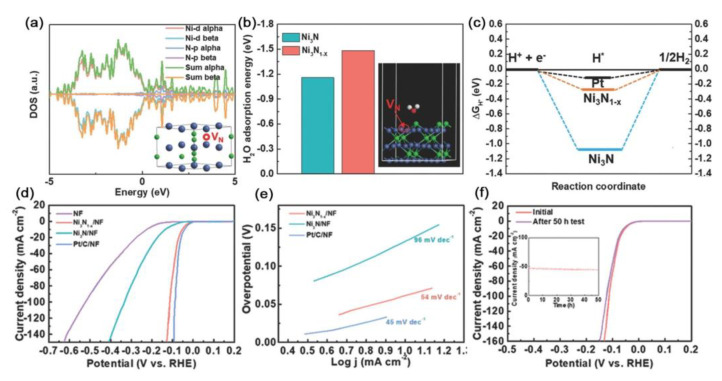
(**a**) The projected electronic TDOS and PDOS of Ni_3_N_1−*x*_. Inset shows the atomic structure model of Ni_3_N_1−*x*_. The horizontal dashed lines represent the Fermi level (0 eV). (**b**) Partial charge density distribution of Ni_3_N_1−*x*_. (**c**) The calculated free-energy diagram of HER. (**d**) HER polarization curves measured in 1.0 M KOH solution (pH 14). (**e**) Corresponding Tafel plots. (**f**) LSV curves before and after the stability test for 50 h. Inset shows the chronoamperometry curve. Reproduced with permission from ref. [94]. Copyright 2020, Wiley-VCH.

Furthermore, through the facile strategy of nitrogen plasma, Liu et al. prepared an unconventional nickel nitride nanostructure enriched with nitrogen vacancies (Ni_3_N_1−*x*_) and systematically investigated the effect of nitrogen vacancies on the HER performance by using first-principles calculations [94]. Density functional theory (DFT) calculations revealed that the downshifting of the d-band center in Ni_3_N_1−*x*_ relative to that in Ni_3_N due to the presence of nitrogen vacancies facilitated the desorption of hydrogen from its surface (Figure 3a). 

In addition, the higher adsorption energy (absolute value) of Ni_3_N_1−*x*_ enriched with nitrogen vacancies than that of the stoichiometric Ni_3_N indicated its superior H_2_O adsorption capability (Figure 3b). Furthermore, Ni_3_N_1−*x*_ with nitrogen vacancies had a considerably lower surface |ΔG_H*_| value than Ni_3_N that contributed to the boosted adsorption–desorption behavior of the intermediately adsorbed hydrogen H* and thus proved its better activity toward HER (Figure 3c). The decline in the Tafel slope of Ni_3_N_1−*x*_ after the introduction of nitrogen vacancies was indicative of the considerably accelerated reaction kinetics of water splitting. As a result, Ni_3_N_1−*x*_ exhibited superior HER performance with 55 mV to achieve the overpotential of 10 mA cm^−2^ and the low Tafel slope of 54 mV dec^−1^ (Figure 3d,e). Its electrocatalytic activity can be well maintained for at least 50 h (Figure 3f).

### 3.2. Strain Engineering

In addition to defects/vacancies, the d-band widths and d-band center can be regulated by using the lattice strain in a single compound as a tuning knob to enhance electrochemical reactions [95,96,97,98,99,100]. Lattice strain can be introduced through lattice mismatch, substrate induction, and heteroatom substitution and is usually accompanied by lattice distortions and rich defects [96]. 

**Figure 4:**
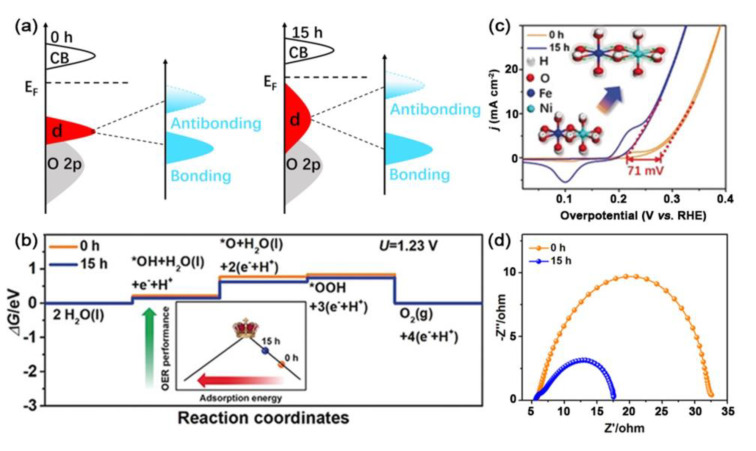
(**a**) Schematic illustration of the band structure of NiFe−LDH. (**b**) The calculated Gibbs free energy of OER for NiFe−LDH. (**c**) CV curves after *i*R and BET−correction. (**d**) Nyquist plots of NiFe−LDH. Reproduced with permission from ref. [99]. Copyright 2019, Wiley-VCH.

By using an easy ball-milling method, Zhou et al. enhanced the binding strength of NiFe hydroxide to oxygenated intermediates via generating tensile strain and then estimated its electrocatalytic OER performance [99]. DFT calculations revealed that introducing tensile strain into NiFe–LDH upshifted the d-band center toward the Fermi level, therefore causing the less filled anti-bonding state and a narrow band gap (Figure 4a).

The adsorption energies for oxygenated intermediates on ball-milled NiFe–LDH were increased through the introduction of tensile strain, thus proving the fast kinetics of this material for the OER process (Figure 4b). In addition, after introducing tensile strain into NiFe–LDH, the Gibbs free energy for every elementary step was optimized, and the energy barrier was significantly reduced, indicating its excellent potential as an OER electrocatalyst. With the increase in tensile strain after ball-milling, NiFe–LDH exhibited low charge transfer resistance (*R*_ct_) and fast reaction kinetics, a relatively low OER onset potential of 1.44 V, and the overpotential of 270 mV vs. RHE for achieving 10 mA cm^–2^ (Figure 4c,d).

**Figure 5:**
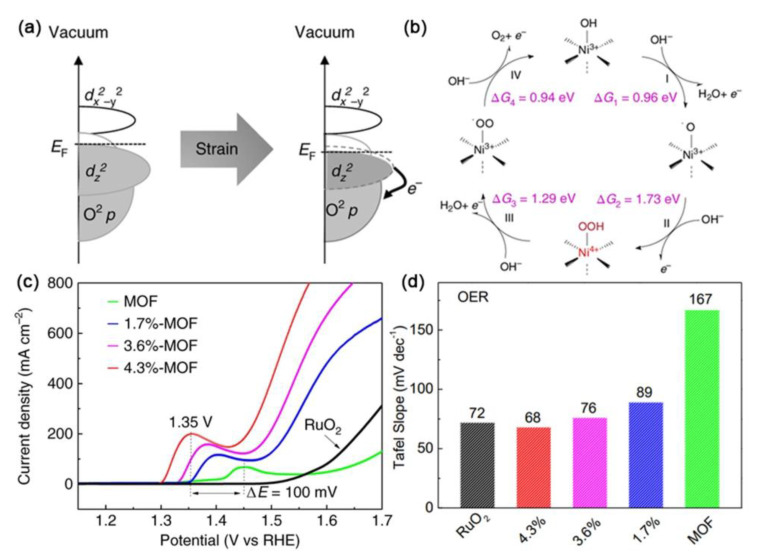
(**a**) Schematic illustration of the electron exchange for the lattice−strained NiFe MOF. (**b**) Proposed OER catalytic mechanisms for the lattice-strained NiFe MOF. (**c**) LSV curves and (**d**) Tafel slope values of OER for the pristine, 1.7%−, 3.6%− and 4.3%−NiFe MOFs. Reproduced with permission from ref. [101]. Copyright 2019, Springer Nature.

Through a facile and controllable process to photoinduce lattice strain, Cheng et al. prepared lattice-strained NiFe MOFs as efficient oxygen electrocatalysts [101]. DFT-based band structure calculations showed that the Fermi level negatively shifted toward the occupied 3*d* bands of nickel with the increase in lattice strain. This shift potentially promoted electron exchange and led to increased covalency in the Ni–O bond (Figure 5a). Further analysis revealed that due to the applied tensile lattice strain, catalytic kinetics transformed from a low-efficiency catalytic process into a fast and efficient 4e^−^ catalytic process. The emergence of Ni^4+^ in the lattice-strained NiFe MOF resulted in the generation of surface superoxide intermediates, which contributed to the high-efficiency of 4e^−^ oxygen electrocatalysis (Figure 5b). As a result, the lattice-strained 4.3%-NiFe MOF showed a relatively low overpotential of ~210 mV at 200 mA cm^−2^ with a Tafel slope of 68 mV decade^−1^ (Figure 5c,d).

### 3.3. Element Doping and Element Substitution

Highly active electrocatalysts for effective water splitting can be developed effectively through tuning the d-band center positions via element doping and element substitution into specific iron-series compounds [102,103,104,105]. 

**Figure 6:**
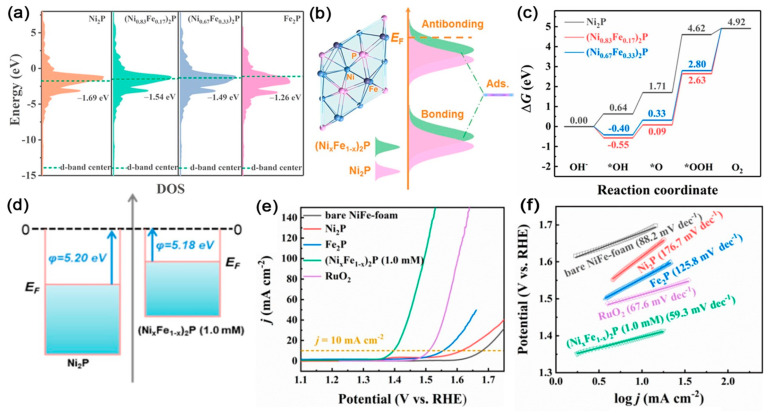
(**a**) d−band centers. (**b**) Schematic illustration of bond formation between the reaction surface and the adsorbate. (**c**) The calculated free−energy (eV) diagram. (**d**) Schematic of work functions. (**e**) LSV curves and (**f**) Tafel plots of the prepared samples. Reproduced with permission from ref. [106]. Copyright 2020, American Chemical Society.

Chen et al. prepared Fe-substituted Ni_2_P ((Ni*_x_*Fe_1–*x*_)_2_P) nanosheets on NiFe foam and evaluated their performance in electrocatalytic OER [106]. DFT calculations illustrated that the *E*_d_ energy level of (Ni*_x_*Fe_1–*x*_)_2_P had increased compared with that of single Ni_2_P. This increment significantly strengthened the interaction between adsorbates and the electrocatalyst surface and thus enhanced the adsorption ability for intermediates (*O, *OH, and *OOH) during the OER process in an alkaline electrolyte. The considerably reduced adsorption free energies of all intermediates after iron substitution were indicative of superior capability for water splitting (Figure 6a–c). Moreover, (Ni*_x_*Fe_1–*x*_)_2_P had a low work function (φ), which demonstrated that it had a weakened electron binding restriction capacity that promoted electrons to escape from the material surface and participate in the catalytic reactions (Figure 6d). The (Ni*_x_*Fe_1–*x*_)_2_P electrocatalyst showed stable OER activity in 1.0 M KOH with the overpotential of only 166 mV at the current density of 10 mA cm^−2^ and a lower Tafel slope of 59.3 mV dec^−1^ (Figure 6e,f). 

**Figure 7:**
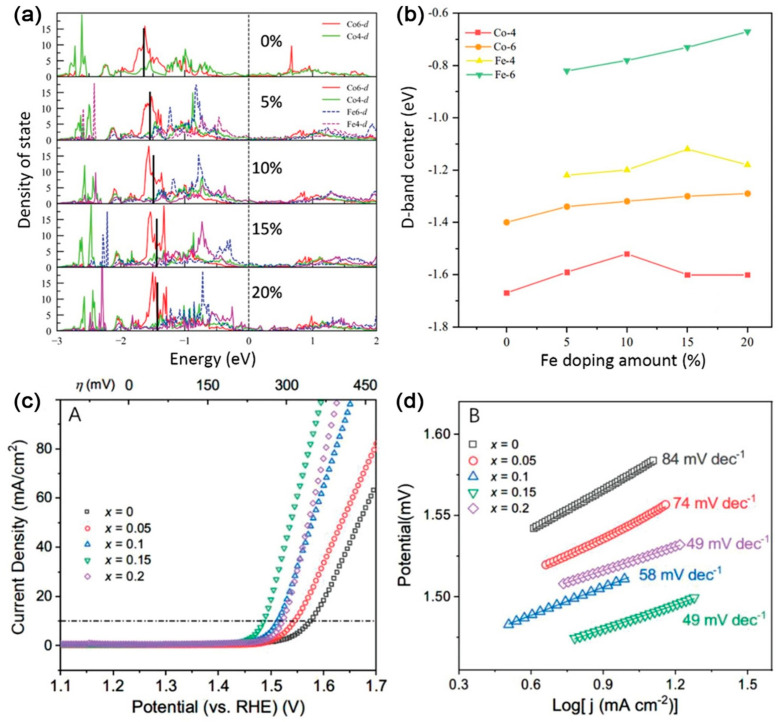
(**a**) Density of states of (Co_1−*x*_Fe*_x_*)_9_S_8_ for *x* = 0, 0.05, 0.1, 0.15, 0.2, respectively. The black solid line shows the d-band center of the bulk phase. (**b**) The d-band center of Fe and Co in various polyhedrons coordinated with 4 or 6 S atoms. (**c**) *i*R−corrected linear sweep voltammetry curves. (**d**) Tafel plots of the prepared samples. Reproduced with permission from ref. [107]. Copyright 2020, Wiley-VCH.

Through an Fe-incorporated topochemical deintercalation method, Zhong and co-workers redesigned the polyhedrons in Co_9_S_8_ to regulate the d-band center [107]. DFT calculations were performed on the band structure and reaction energy profile of the catalyst, and their corresponding results showed that the d-band center was gradually upshifted when the doping amount of heteroatomic iron was increased. This change contributed to aiding the adsorption of reaction radicals (Figure 7a). In addition, the summarized d-band centers of the corresponding Fe/Co tetrahedrons and octahedrons showed that the six-coordinated iron octahedron exhibited a high d-band center that was upshifted relative to the Fermi level with the amount of iron content, implying that the elevation of the overall d-band can be mainly due to the iron octahedrons (Figure 7b). Benefitting from these features, the best sample of Fe–0.15–Co_9_S_8_ exhibited superior OER activity over the other as-prepared samples and displayed an overpotential of 255 mV at the current density of 10 mA cm^−2^ and a Tafel slope of 49 mV dec^−1^ (Figure 7c,d).

Furthermore, Wang et al. successfully manipulated Co_4_N nanosheets (NSs) for HER catalysis through tailoring their d-band centers by doping with the transition metal V [108]. 

**Figure 8:**
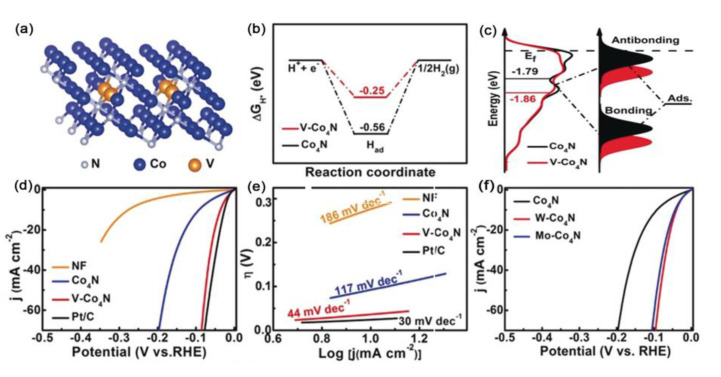
(**a**) The geometric configuration of V−Co_4_N (111) with V atoms replacing the subsurface Co atoms. (**b**) Free−energy diagram for the HER. (**c**) The density of states (DOS) plots as well as the corresponding schematic illustration of bond formation between the catalyst surface and the adsorbates. (**d**) LSV curves and (**e**) the Tafel slopes of the prepared samples. (**f**) The LSV curves of Co_4_N, W−Co_4_N, and Mo−Co_4_N. Reproduced with permission from ref. [108]. Copyright 2018, Wiley-VCH.

DFT calculations revealed that the free energy of the adsorbed hydrogen (Δ*G*_H*_) on V–Co_4_N was closer to the thermoneutral value than that of Co_4_N (−0.56 eV), suggesting that hydrogen adsorption/desorption was aided (Figure 8a). Moreover, after V doping, the d-band center became distant from the Fermi level, thus decreasing the adsorption energy of hydrogen and facilitating hydrogen desorption from the catalyst surface for HER catalysis (Figure 8b). As a result, the V–Co_4_N NSs exhibited superior electrocatalytic performance with the overpotential of 37 mV at 10 mA cm^−2^ and a relatively low Tafel slope of 44 mV dec^−1^ (Figure 8c,d). Importantly, W and Mo doping had been verified to exhibit similar behaviors in tuning the positions of the d-band center (Figure 8e). 

**Figure 9:**
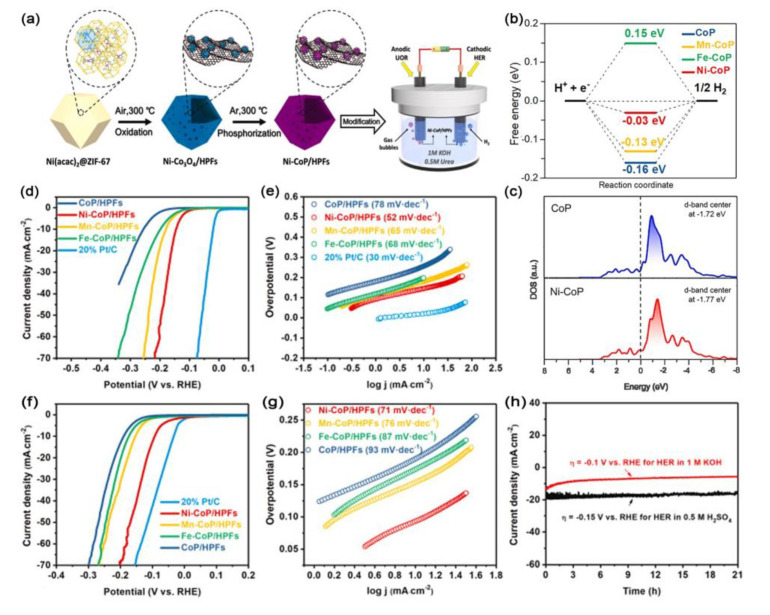
(**a**) Synthesis scheme of the Ni-CoP/HPFs. (**b**) The calculated free-energy diagram of Ni−CoP, Mn−CoP, Fe−CoP, and CoP. (**c**) Calculated DOS curves for CoP and Ni−CoP. (**d**,**f**) LSV curves, (**e**,**g**) Tafel plots in 0.5 M H_2_SO_4_ and 1 M KOH, respectively. (**h**) Time-dependent of current density curves over Ni−CoP/HPFs catalyst during electrolysis at −0.15 V vs. RHE in 0.5 M H_2_SO_4_ and −0.1 V vs. RHE in 1 M KOH [109]. Copyright 2019, Elsevier B.V.

Chen et al. prepared M-doped CoP (M = Ni, Mn, Fe) HPFs catalysts for HER in both acid and alkaline media through the self-templating transformation strategy (Figure 9a) [109]. As revealed by XANES, XPS, AES, UPS, and DFT calculations, the d-band center of the M-CoP/HPF catalyst was downshifted away from the Fermi level relative to that of its counterparts. This result suggested that the interaction of metal-P caused the change in the valence band structure of M-CoP/HPFs. The downshifted d-band center of Ni-CoP/HPFs decreased the adsorption energy of hydrogen, and thus helped the desorption of hydrogen from the surface of the M-CoP/HPFs for HER (Figure 9b,c). As expected, the Ni-CoP/HPFs exhibited excellent catalytic activity with the overpotentials of 144 mV (0.5 M H_2_SO_4_) and 92 mV (1 M KOH) to achieve a current density of 10 mA cm^−2^ in HER, and excellent robustness with slight variations after a 21 h long-term stability test (Figure 9d–h).

**Figure 10:**
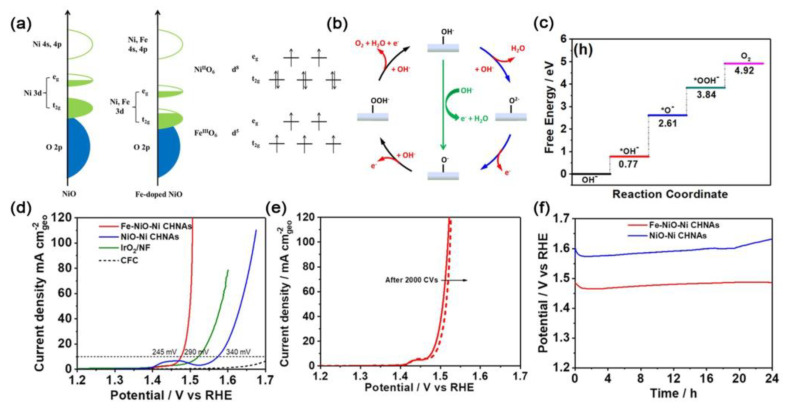
(**a**) Schematic diagram of the band structure of Fe−NiO−Ni CHNAs and NiO−Ni CHNAs. (**b**) Proton−electron transfer pathway and nonconcerted proton–electron transfer pathway of OER. (**c**) Free-energy diagrams of OER for Fe−doped NiO with proton−electron transfer pathway. (**d**) LSV curves of the prepared samples in 1 M KOH at a scan rate of 5 mV s^−1^ after *i*R correction. (**e**) LSV curves of Fe−NiO−Ni CHNAs before and after 2000 CVs. (**f**) E-t curves of Fe-NiO−Ni CHNAs and NiO−Ni CHNAs at current density of 10 mA cm^−2^ [110]. Copyright 2020, Elsevier B.V.

In another example, Sun and co-workers recently reported that Fe-doped NiO coupled nickel cluster hollow nanotube arrays (Fe–NiO–Ni CHNAs) prepared through the in situ anodic oxidation strategy are efficient OER catalysts [110]. X-ray absorption fine structure revealed that the pre-edge of the O k-edge in Fe–NiO–Ni CHNAs was negatively shifted, which indicated that the Fe-doping downshifted the d-band (Figure 10a). The downshifting of the d-band center of M sites can reduce the adsorption energy of the intermediates and facilitate OER kinetics, thus causing the Ni/Fe 3d and O 2p centers in Fe-NiO-Ni CHNAs to move close to each other and leading to the increase in M–O covalency (Figure 10b). The increased M–O covalency can accelerate electron transfer between M cations and O adsorbates, and help the extraction of electrons from oxygen, thereby greatly promoting the OER process (Figure 10c,d). As a result, the Fe–NiO–Ni CHNAs electrocatalyst presented the overpotential of 245 mV to deliver the current density of 10 mA cm^−2^, and to exhibit excellent stability for over 24 h that surpassed the stability of most transition metal oxides (Figure 10e,f).

### 3.4. Alloying

Alloying multiple iron-series elements to construct specific nanostructures is also an effective method for improving the catalytic activities of materials by modulating electronic structures through tuning the position of the d-band center [111,112,113,114]. The incorporation of one iron-series transition metal into another iron-series transition metal can modify electronic structures. In view of this, a monolithic alloy was prepared to improve the OER activity of single iron-series transition metal catalysts [115,116,117,118]. 

**Figure 11:**
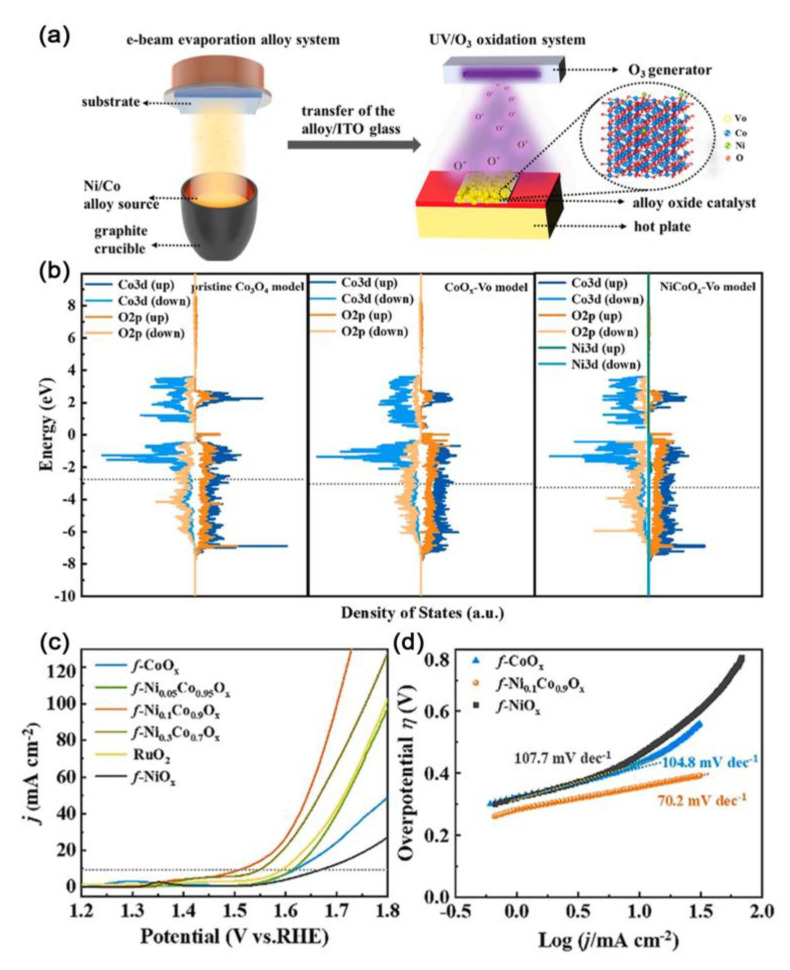
(**a**) Schematic illustration of the synthetic process of *f*−Ni_0.1_Co_0.9_O_x_ film. (**b**) The diagram of PDOS. Electrochemical OER performance assessments without any ohm compensation. (**c**) Polarization curves of Ni/Co alloy oxides. (**d**) Tafel plots. Reproduced with permission from ref. [119]. Copyright 2021, Elsevier B.V.

Ma and coworkers proposed an e-beam evaporation alloy-UV/O_3_ oxidation method for the fabrication of optically transparent NiCo bimetallic alloy oxide electrocatalysts for OER in 1.0 M KOH electrolyte through introducing the nickel heteroatom into CoO_x_ (Figure 11a) [119]. Experimental and theoretical calculations confirmed that the oxygen vacancy concentration can be regulated by changing the Ni/Co proportion of the alloy to obtain additional active sites on the surfaces and edges of the defective spinel structure. DFT-based calculations demonstrated that the d-band center of cobalt in the structure of the NiCoO_x_-Vo model was below the Fermi level and had a lower energy than that of the CoO_x_-Vo model and the pristine Co_3_O_4_ model. These characteristics indicated that the NiCoO_x_-Vo model had suitable adsorption for oxygen species (Figure 11b). As a result, *f*-Ni_0.1_Co_0.9_O_x_ exhibited excellent OER performance with the ultrahigh catalytic mass activity of 3055 A g^−1^ at the overpotential of 250 mV and a Tafel slope of 70.1 mV dec^−1^, thus presenting a mass activity that was almost 190 and 7.5 times higher than the mass activities of commercial RuO_2_ and *f*-CoO_x_, respectively (Figure 11c,d).

Furthermore, Li et al. boosted the intrinsic OER activity of Co-based bimetallic nanoparticles by incorporating them into alloys through a melamine bridged self-construction strategy [120]. 

**Figure 12:**
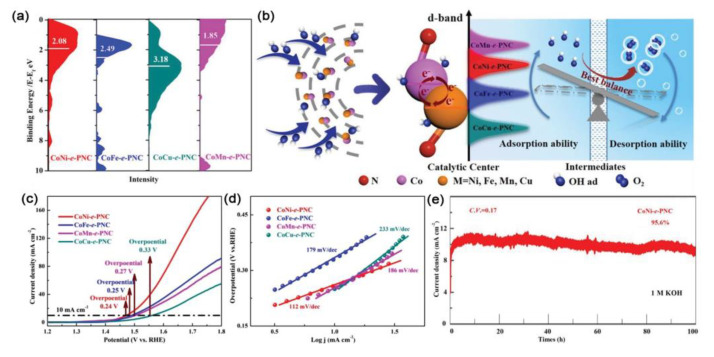
(**a**) Valence band spectra (the white bar shows d-band center). (**b**) Schematic illustration for the OER reaction process of CoM−*e*−PNC catalysts. (**c**) Polarization curves. (**d**) Tafel plots. (**e**) *i*−*t* curves obtained at 10 mA cm^−2^ for CoNi−*e*−PNC catalyst. Reproduced with permission from ref. [120]. Copyright 2019, Wiley-VCH.

It should be noted that the active substances used to compare the d-band center with each other here are compounded with N-doped carbon sphere, thus excluding the influence of carbon on the d-band center, which is only affected by alloying. The calculation results for the d-band center of the prepared samples indicated that the position of the d-band center sites could be controllably tailored through the alloying of cobalt and another transition metal M (M = Ni, Fe, Mn, and Cu) (Figure 12a). The balance between the adsorption of OH species and the desorption of O_2_ was thus altered by the changes in the d-band center sites of Co-based bimetallic nanoparticles, eventually improving the intrinsic OER activity of CoM (Figure 12b). Through association with the above unique open hierarchical pore structure, the CoNi-*e*-PNC catalyst presented optimal OER performance with the overpotential of 240 mV and demonstrated high electrocatalytic activity for up to 100 h at 10 mA cm^−2^ in alkaline solutions (Figure 12c–e).

### 3.5. Composite of Two or More Iron Transition Series Metal-Based Compounds

In addition to that of catalysts consisting of an iron-series metal and a compound based on an iron-series metal, the investigation of materials that consist of two or more iron transition series compounds as efficient electrocatalysts for water splitting has attracted great interest. 

Through typical hydrothermal and electrodeposition methods, Zhang et al. prepared a series of Ni_3_S_2_@MOOH/NF (M = Fe, Ni, Cu, Mn, and Co) hybrid structures with enhanced HER performance [121]. Various spectral analysis and DFT calculations indicated that the d-band center of the Ni_3_S_2_@NiOOH heterogeneous interface had moved slightly to the left compared with that of Ni_3_S_2_ and was slightly shifted to the right relative to that of NiOOH. These shifts were indicative of the weakened binding of the adsorbed hydrogen on the catalytic site and the optimized binding energy of the active site to H* at the interface of the Ni_3_S_2_@NiOOH heterogeneous structure. 

**Figure 13:**
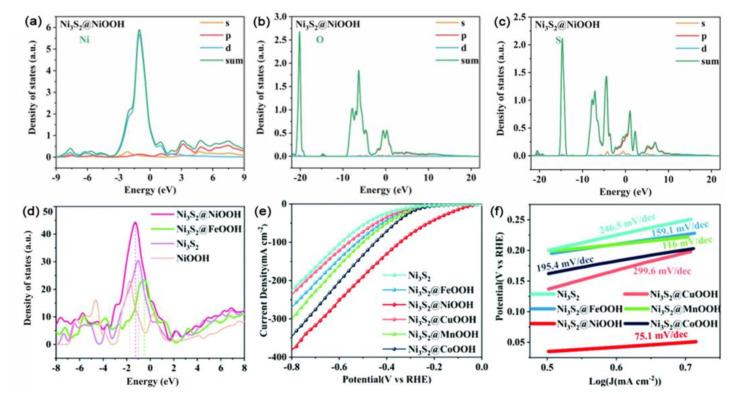
Density of states for Ni_3_S_2_@NiOOH, (**a**) Ni, (**b**) O, and (**c**) S. (**d**) Density of states of H_2_O for Ni_3_S_2_, NiOOH, Ni_3_S_2_@NiOOH, and Ni_3_S_2_@FeOOH. (**e**) Polarization curves for HER at a scan rate of 5 mV s^−1^ in 1.0 M KOH and (**f**) the corresponding Tafel plots [121]. Copyright 2021, The Royal Society of Chemistry.

These effects contributed to the improved catalytic activity and optimized electronic structure of Ni_3_S_2_@NiOOH (Figure 13a,b). As a result, the synthesized Ni_3_S_2_@NiOOH core–shell structure presented good HER performance in alkaline media with the overpotential of 79 mV at the current density of 10 mA cm^−2^ and a significantly low Tafel slope of 75.1 mV per dec^−1^, which is one of the best catalytic activities reported so far (Figure 13c,d).

Gao et al. carried out a simple solvothermal method to design self-supported NiO/Co_3_O_4_ heterogeneous structures for the OER [122]. DFT calculations were performed to calculate the d-band centers of the cobalt atoms at different positions to study the effects of heterojunctions. 

**Figure 14:**
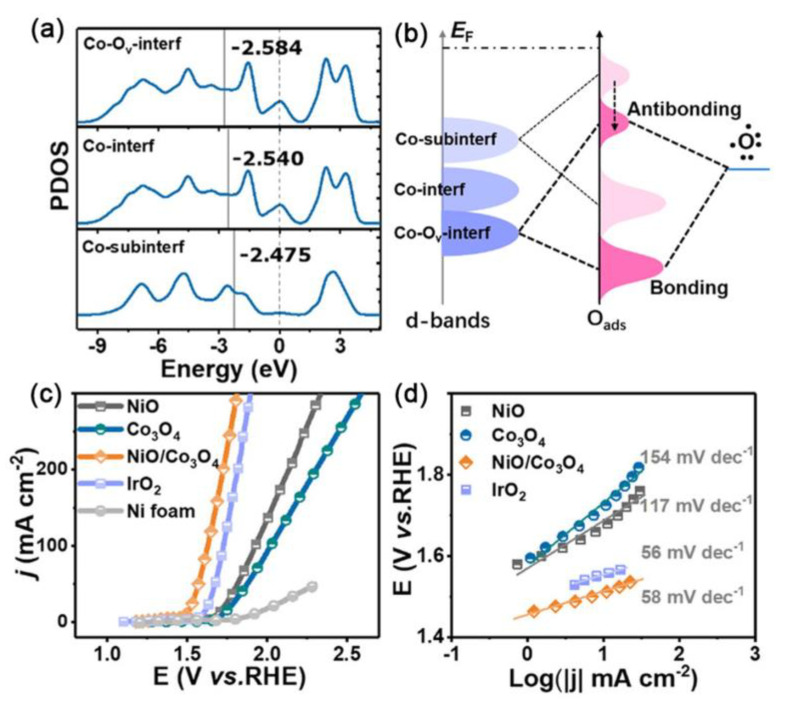
(**a**) PDOS of Co−3d bands at different positions. (**b**) Schematic bond formation of atomic oxygen on shifting down d-band centers of Co. (**c**) Polarization curves after *i*R−correction, commercial IrO_2_ and Ni foam were used for comparison. (**d**) Corresponding Tafel slopes obtained from polarization curves [122]. Copyright 2020, American Chemical Society.

The d-band center of the interfacial cobalt was lower than that of sub-interfacial cobalt in NiO/Co_3_O_4_, and the d-band center of cobalt at the heterointerface further shifted negatively in the NiO/Co_3_O_4_–O_v_ sample (Figure 14a,b). Therefore, the cobalt atoms at the interface with the lower d-band center may exhibit weaker and more suitable adsorption for oxygen species than other atoms, thereby accelerating the kinetics and enhancing the catalytic activity for OER. In experiments, the NiO/Co_3_O_4_ heterostructures presented a relatively low overpotential of 262 mV at the current density of 10 mA cm^−2^ with a low Tafel slope of only 58 mV dec^−1^ for the OER (Figure 14c,d).

## 4. Conclusions and Future Perspectives

The development of electrocatalyst design for clean energy conversion has gained extensive attention from researchers over the last decade. Given that iron, cobalt, or nickel elements can form a variety of oxides or hydroxides with different crystalline phases, and selenides, sulfides, nitrides, and phosphates with different element ratios, they provide a material basis for regulating the electronic structure of the catalyst interface through recombination between different compounds. 

The interfacial properties of compounds based on iron-series metals can be changed due to the following factors: The interaction between anions and metal cations in different compounds based on iron-series metals changes the density of the interfacial electron cloud; the difference of work functions can lead to the movement of electrons at the interface. Recombination leads to the generation of a large number of defects and the transformation from crystalline materials into amorphous materials, resulting in the reduction of atomic valence at the interface. It can be seen that the electron movement at the interface after the recombination contributes to changing the properties of the interface. That is, the electron movement leads to the changes in the valence state, electron cloud density, and the orbital filling degree of the atom, thus resulting in the positive or negative movement of the d-band center of the catalytically active atom.

The d-band theory states that the upshifting of the d-band center promotes hydrogen adsorption and that the downshifting of the d-band center facilitates hydrogen desorption. Therefore, the band center has been confirmed as an efficient descriptor for boosting the performance of electrocatalytic water splitting. Many powerful strategies have been employed in boosting the catalytic performance of electrocatalysts through tuning the d-band center of the transition metals. These strategies include (i) introduction of defects/vacancies, (ii) strain engineering, (iii) element doping/substitution, (iv) alloying, and (v) composites of two or more iron transition series metal-based compounds. These methods may have different regulation effects on the d-band center in different chemical environments, that is, the upshifting of the center is close to the Fermi energy level, and the downshifting of the center is far away from the Fermi energy level. However, whether the center rises or falls, it will be regulated by the experimental methods, which is beneficial to the ultimate catalytic activity. It can be said that for different types of catalysts, there is a suitable position for the d-band center. Although the above strategies have exhibited high potential and enable HER or OER in acid or alkaline solutions with very low overpotential and good stability, several issues need to be taken into consideration for stable and economical operation. In addition, the geometrical area of the electrode cannot fully reflect the actual electrochemical active area of the three-dimensional electrode, which may cause some errors when evaluating the d-band effect. Therefore, the electrochemical active surface area rather than the geometric area is the most important and accepted to objectively reflect the impact of d-band regulation when normalizing the current density.

In addition, all of these d-center metals/compounds along with the HER and OER parameters mentioned in this manuscript have been summarized in Table 1.

It can be seen from Table 1 that the effective regulation of the d-band center can be achieved by changing the chemical environment of iron-series metal atoms, and the position of the d-band center is related to the preparing method and element types. The d-band center needs to be adjusted to an appropriate position to play an optimal electrocatalytic activity.

Recently reported articles have shown that the shift of the d-band center only offers a qualitative explanation for the strengthening or weakening of the binding ability of the key intermediates on the surfaces of electrocatalysts. Therefore, a precise algebraic expression of the relationship between the descriptor and the intrinsic activity of the electrocatalysts is needed for the further design of electrocatalysts with the desired activity and stability. However, only a single descriptor is often proposed for the prediction of the electrocatalytic activity of the catalysts. This situation may run into failure under certain circumstances. Given the complexity of electrochemical systems, a multidimensional descriptor matrix that includes multiple physicochemical properties of the materials may be the solution to this issue. Therefore, the descriptor of the d-band center should be combined with other parameters, such as the pH value, hydrogen-bonding strength, elemental valences, and interfacial water, to describe the mechanism underlying electrochemical catalysis with increased comprehensiveness.

## Figures and Tables

**Table 1:** Relationships between the d-band center and catalytic performances of catalysts using different regulation methods.

Catalysts	D-Band Center	HER (at 10 mA cm^−2^)	OER (at 10 mA cm^−2^)	Ref.
NiCo_2_O_4_	downward shift	135 mV in 1 M KOH	240 mV in 1 M KOH	[22]
CoP	upward shift	84 and 94 mV in acidic and alkaline media, respectively	/	[93]
Ni_3_N_1−x_	downward shift	55 mV in 1 M KOH	/	[94]
NiFe–LDH	upward shift	/	270 mV in 1 M KOH	[99]
NiFe MOFs	negatively shifted	/	210 mV at 200 mA cm^−2^ in 0.1 M KOH	[101]
Fe-substituted Ni_2_P	upward shift	/	166 mV in 1 M KOH	[106]
Co_9_S_8_	upward shift	/	255 mV in 1 M KOH	[107]
V–Co_4_N	downward shift	37 mV in 1 M KOH	/	[108]
M-doped CoP	downward shift	144 mV in 0.5 M H_2_SO_4_	92 mV in 1 M KOH	[109]
Fe-doped NiO	downward shift	/	245 mV in 1 M KOH	[110]
NiCo bimetallic alloy oxide	downward shift	/	268 mV in 1 M KOH	[119]
Co-based bimetallic nanoparticles	tailored	/	240 mV in 1 M KOH	[120]
Ni_3_S_2_@MOOH/NF	downward shift	79 mV in 1 M KOH	/	[121]
NiO/Co_3_O_4_	downward shift	/	262 mV in 1 M KOH	[122]

## Data Availability

Not applicable.
